# Consumer or Decomposer? Behavioral and Morphological Diagnosis of White Grubs

**DOI:** 10.1002/ece3.71925

**Published:** 2025-08-11

**Authors:** Yi‐Nuo Chen, Mei Ying, Chang Lu, Lu Jiang

**Affiliations:** ^1^ Key Laboratory of Economic and Applied Entomology of Liaoning Province, College of Plant Protection Shenyang Agricultural University Shenyang Liaoning China

**Keywords:** cockchafer, feeding preference, insect larva, scarab beetle, six‐armed olfactometer

## Abstract

Determining the ecological role of insect larvae is crucial for pest control and resource utilization, which usually relies on accurate species identification. White grubs, the larvae of Scarabaeoidea, exhibit highly diverse behaviors but share a similar morphology, making identification challenging. In this study, an improved six‐armed olfactometer was used to test the feeding preferences of two sympatric white grubs, *Anomala mongolica* and *Protaetia brevitarsis*. The correlated morphological traits, including the sensory, feeding, and locomotive organs, were investigated using cross‐sections and scanning electron microscopy. Our results show that phytophagous grubs possess well‐developed antennal sensilla and digitiform sensilla on maxillary palps, mandibles with incisors for cutting plant roots, and fossorial setae and sharp claws on thoracic legs. In contrast, saprophagous grubs have underdeveloped digitiform sensilla, relatively short and robust antennae and thoracic legs, and mandibles lacking cutting edges suitable for severing roots. This study provides new insights for determining the ecological roles of white grubs.

## Graphical abstract

Determining the ecological role of insect larvae is crucial for pest control and resource utilization, relying on effective behavioral or morphological identification traits. This study uses an improved six‐armed olfactometer to analyze the feeding habits of two sympatric white grubs and the correlated morphological traits, including the sensory, feeding, and locomotive organs, providing new insights for determining the ecological roles of the white grubs.

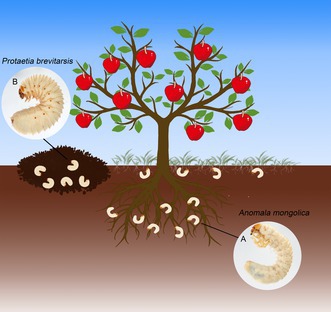

## 1 Introduction

Determining the ecological role of insect larvae is essential for pest control and resource utilization. However, this task is often complicated by morphological homogenization, particularly among soil‐dwelling arthropods (Villani et al. 1999; Wagner 2005), likely driven by convergent selective pressures (Jiang et al. 2019). Even within the highly diverse Scarabaeoidea, adult morphology exhibits considerable variation, while larval morphology tends to be more homogenized (Frew et al. 2016; Eberle et al. 2019).

The larvae of scarab beetles, commonly known as white grubs, are highly recognizable in appearance and have a characteristic C‐shape (Zhang 1984). Many of these grubs are important agricultural pests, causing growers to fear them and resort to the use of broad‐spectrum, highly toxic insecticides for root treatment (Anuar et al. 2010), leading to severe environmental pollution. In fact, white grubs play a very complex role in ecosystems (Li, Fu, et al. 2019; Li, Jiang, and Fang 2019; Wang et al. 2024). Larvae of Geotrupidae, Scarabaeinae, and Aphodiinae are coprophagous, feeding on feces (Li, Fu, et al. 2019; Li, Jiang, and Fang 2019). Whereas the larvae of Cetoniinae and Dynastinae are saprophagous, usually consuming decomposing organic matter (Dong et al. 2021; Jiang et al. 2025). Even among the vast majority of so‐called phytophagous groups, different species of larvae feed on different host plants (Morón et al. 2018; Jia et al. 2023; Cao et al. 2024). Identifying species based on larval morphological characteristics is a necessary prerequisite for both pest control and the utilization of beneficial insect resources. However, the identification of white grub species has always posed significant challenges, with only about 2% of species being directly identifiable (Newton 1990; Lawrence et al. 2011).

Rather than attempting to overcome the great difficulty of identifying individual larvae, can we find a way to determine whether a grub is a pest or a beneficial insect without the need for precise species identification? Actually, many references exist that can help infer morphological traits related to feeding, such as sensory organs for food location (Zacharuk and Shields 1991; Eilers et al. 2012; Lu 2021), feeding structures, locomotion organs, and digestive systems (Wang et al. 2024). However, few morphological studies directly link these traits to specific feeding habits. Several classic methods are used to determine an insect's feeding habits, including leaf disk assays (Ali et al. 1999; Filho and Mazzafera 2000; Shields et al. 2008), Y‐tube bioassays (Lu et al. 2024), gut content analysis (Cooper et al. 2016), weight change measurements (Oonincx et al. 2015), and video monitoring (Itskov et al. 2014). However, these methods are not effective for solving the problem with grubs that are hidden in the soil.

In this study, we used an improved six‐armed olfactometer to analyze the feeding habits of two sympatric white grubs, *Anomala mongolica* and *Protaetia brevitarsis*, aiming to provide evidence of their different feeding behaviors. Optical and scanning electron microscopy are used to examine morphological traits related to feeding, such as sensory organs, feeding structures, and locomotion organs. The main goal is to provide morphological features that help identify the ecological roles of these grubs.

## 2 Materials and Methods

### 2.1 Insect Collection and Rearing

Living adults of *A. mongolica* were collected under light traps, while *P. brevitarsis* were collected using sweeping nets. All specimens of both species were collected in the experimental field at Shenyang Agricultural University, in Liaoning Province, northeast China. After accurate identification by examining the external genitalia following Kim (2011), the copulated adults were reared in plastic boxes, covered with plastic film to retain humidity. The boxes were filled with humid sandy soil to a depth of 10 cm for egg deposition. The environmental temperature was maintained at 25°C. *A. mongolica* adults were fed fresh elm leaves daily, while their larvae were provided with soaked wheat, following the modified rearing methods described by Ritcher (1966) and Jia et al. (2020). *Protaetia brevitarsis* adults were fed bananas, and their larvae were given moist fermented sawdust as their diet, according to the rearing methods described by Dong et al. (2021).

### 2.2 Feeding Preference Experiments

**FIGURE 1:**
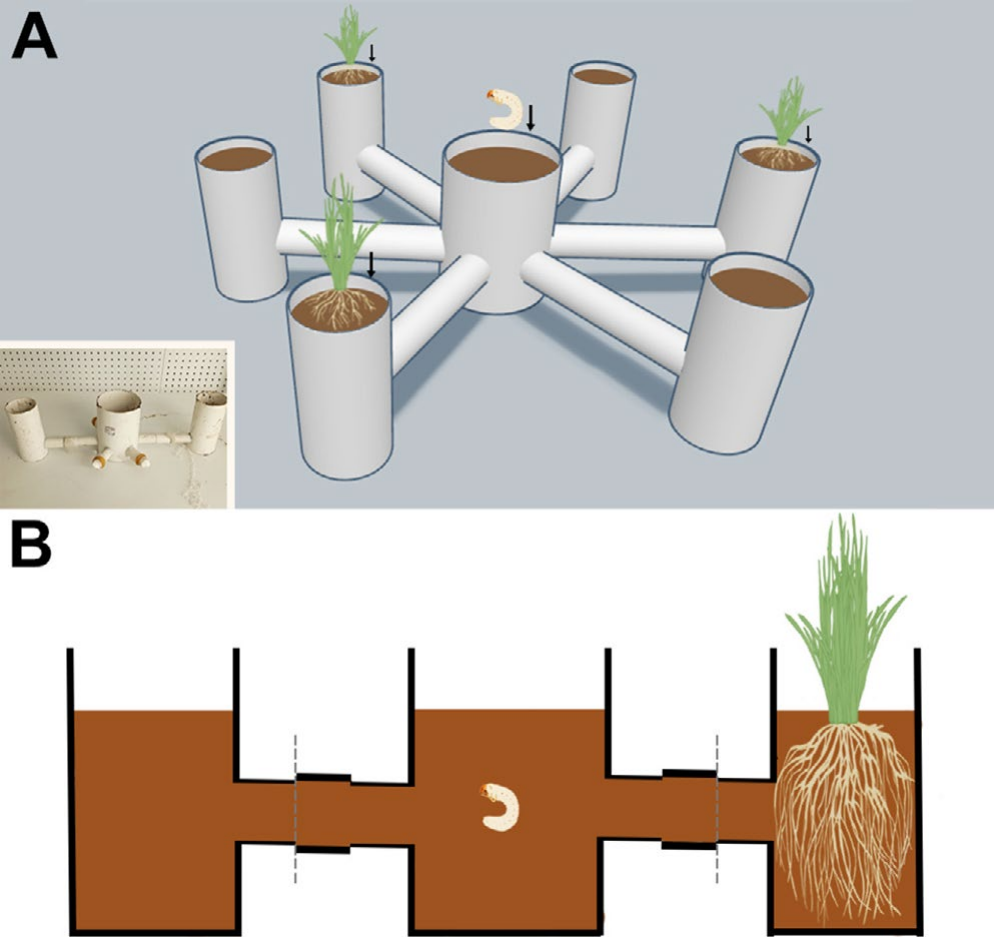
Feeding preference behavior research apparatus. (A) Six‐armed olfactometer; (B) Cross‐sectional view of the six‐armed olfactometer.

To test the feeding preferences of white grubs, we modified a six‐armed olfactometer based on a nematode behavioral model (Rasmann et al. 2005), making it suitable for white grub behavior experiments. The inner diameter of the connecting tubes was set to 3 cm (Figure 1A). During the experiment, moist fermented wood chips were placed to a depth of 18 cm in all six sample chambers. In three alternating sample chambers, 20 wheat plants were planted 20 days prior to the experiment, allowing their root systems to fully develop within the chambers. The opposite chambers were left without wheat as controls (Figures S1 and S2). At the start of the experiment, 10 third‐instar larvae of each species (*n* = 10), which had been starved for 24 h, were introduced into the central chamber of the six‐armed olfactometer (Figure 1B). After 24 h, the positions of the larvae were observed and recorded (Figures [Link], [Link], [Link], [Link]). The entire experiment was repeated three times (*N* = 3) under controlled environmental conditions: a temperature of 21°C, relative humidity of 65%, and a photoperiod of 16L:8D. The data were analyzed using SPSS 25.0 with chi‐squared tests to compare larval distribution between treatment and control chambers. A preference index (PI) was calculated as PI = (Np‐Nc)/(Np + Nc), where Np and Nc represent the number of larvae in plant and control chambers, respectively. One‐sample *t*‐tests were used to determine if PI values significantly differed from zero (*p* < 0.05). Statistical analyses were conducted in SPSS v25.0 (IBM Corp., Armonk, NY, USA), and results are presented as mean ± standard error (SE), following the methods of Turlings et al. (2012).

### 2.3 Light and Scanning Electron Microscopy

For morphological comparison, at least 10 larvae were fixed in Dietrich's solution (formalin: 95% ethanol: glacial acetic acid: distilled water = 6:15:1:80, v/v). The solution containing the larvae was heated to 70°C, left to stand under a cover for 12 h, and then preserved in 75% ethanol (Jiang and Hua 2015). Photographs were captured using a Nikon D810 digital camera (Nikon Corporation, Tokyo, Japan).

More than 10 specimens of each species were prepared and examined using scanning electron microscopy (SEM) to eliminate potential individual variations. The larvae were dissected under a Leica EZ4HD stereoscopic zoom microscope while submerged in a 70% ethanol solution. The dissected specimens were ultrasonically cleaned for 2 min, rinsed twice, and dehydrated sequentially in graded ethanol. They were then treated with tertiary butanol, freeze‐dried for 3 h, sputter‐coated with gold, and examined under a Hitachi S‐3400N scanning electron microscope (Hitachi, Tokyo, Japan) at 5 kV.

For cross‐sectional observations, the maxillary palps of the larvae were sequentially immersed in alcohol solutions of 75%, 50%, and 25% concentrations, followed by distilled water, with a 30‐min interval at each step to ensure thorough dehydration. A gradient replacement process was then performed to transition from distilled water to the 100% cryo‐section embedding medium. This process involved three steps, using mixtures of distilled water and embedding medium at volume ratios of 3:1, 2:2, and 1:3, with a 1‐h equilibration interval for each. The samples were incubated overnight in the embedding medium. The following morning, sections were prepared using a cryo‐microtome. The sections were mounted with coverslips and baked at 55°C for 30 min in a drying oven. Finally, the prepared samples were observed under a microscope, and images were captured.

## 3 Results

### 3.1 Habitat and Feeding Habits

**FIGURE 2:**
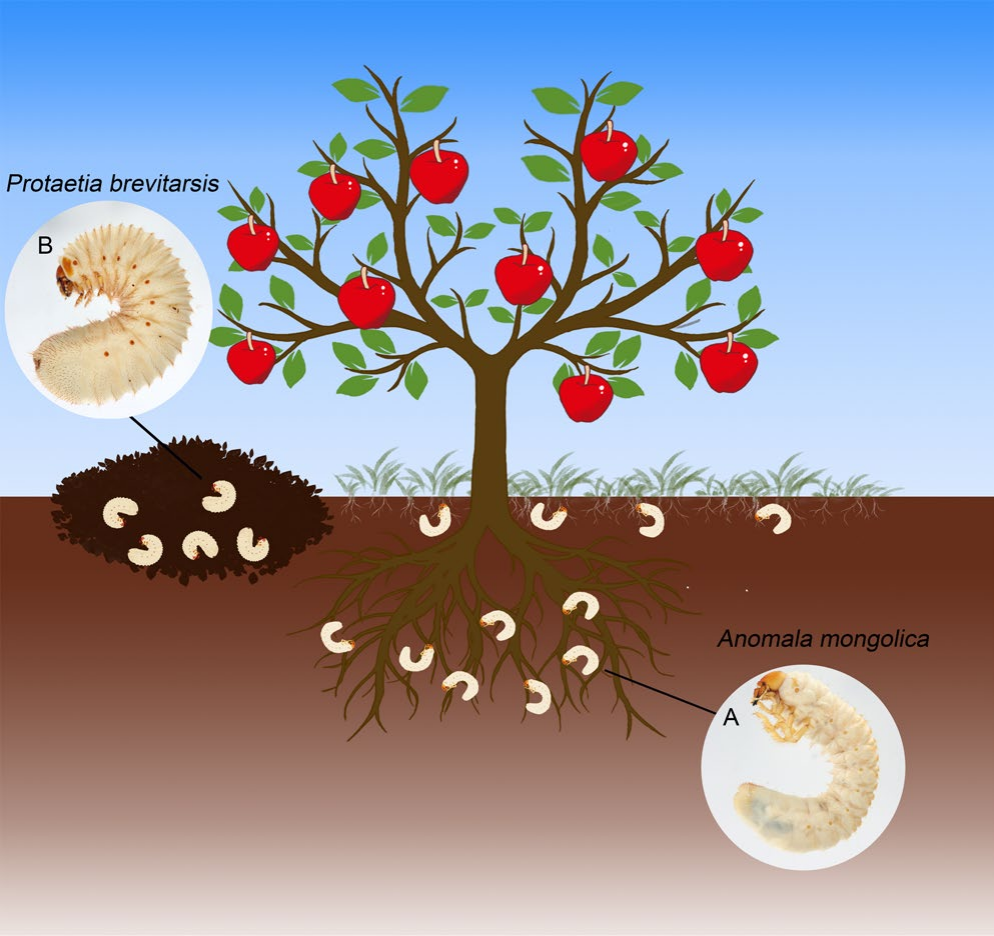
Habitat of the larvae of *Anomala mongolica* and *Protaetia brevitarsis*. (A) *A. mongolica*; (B) *P. brevitarsis*.

Based on our field collections and observations, the larvae of *A. mongolica* and *P. brevitarsis* exhibited notable differences in their habitats (Figure 2). The larvae of *A. mongolica* are typically found around the roots of trees and grasses (Figure 2A), while *P. brevitarsis* larvae prefer compost, decaying grass piles, manure, and humus‐rich soil (Figure 2B). These distinct habitat differences suggest marked differences in feeding behavior between the two species.

### 3.2 Feeding Preference

**FIGURE 3:**
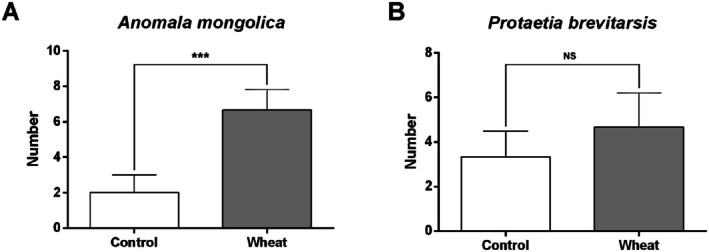
The feeding preferences of the two white grubs. (A) Results of odor tendency test of larvae of *A. mongolica*. (B) Results of odor tendency test of larvae of *P. brevitarsis*. *** indicates the significant difference (*p* < 0.001) in the *t*‐test.

In this experiment, wheat was used to test the attraction of two different species of scarab larvae to plant roots (Figure 3). In the experiment (*N* = 3, *n* = 10 per replicate), 6.67 ± 0.67 larvae of *A. mongolica* were found near wheat, significantly more than the 2.00 ± 0.58 individuals found in the control chambers (*χ* ^2^ = 4.829, *p* = 0.028). The preference index for *A. mongolica* was significantly positive (PI = 0.538 ± 0.089, *t* = 6.057, *p* = 0.026), clearly indicating a preference for plant roots. For *P. brevitarsis* , 3.33 ± 0.67 larvae were found near wheat, while 4.67 ± 0.88 individuals were found in the control chambers, showing no significant difference (*χ* ^2^ = 0.644, *p* = 0.422). The preference index was not significantly different from zero (PI = −0.167 ± 0.153, *t* = −1.091, *p* = 0.389), demonstrating that *P. brevitarsis* larvae exhibited no specific preference for plant roots.

### 3.3 Morphological Differences of Sensing Organs

**FIGURE 4:**
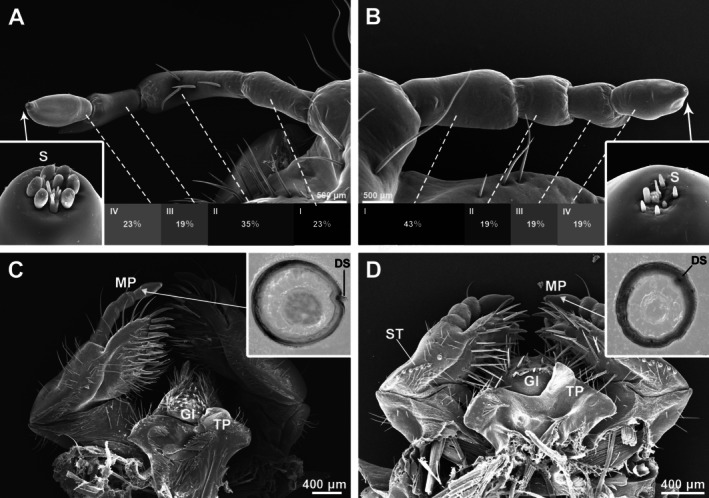
The sensing organs of *Anomala mongolica* and *Protaetia brevitarsis*. (A) Magnification on the antenna of *A. mongolica*; (B) Magnification on the antenna of *P. brevitarsis*; (C) Maxilla, labia, and hypopharynx of *A. mongolica*; (D) Maxilla, labia, and hypopharynx of *P. brevitarsis*. DS, digitiformia sensilla; Gl, glossa; MP, maxillary palp; S, sensillum; ST, stridulatory teeth; TP, truncate process.

The sensory organs of white grubs are distributed on the head capsule, including the antennae and maxillary palps (Figure 4).

The antenna of *A. mongolica* larvae is slender and composed of four segments, measuring sequentially 0.64 ± 0.05 mm (*N* = 10), 0.98 ± 0.06 mm (*N* = 10), 0.57 ± 0.08 mm (*N* = 10), and 0.64 ± 0.05 mm (*N* = 10), accounting for approximately 23%, 35%, 19%, and 23% of the total antennal length, respectively (Figure 4A). The second segment has four setae, and the ventral surface at the front end of the third segment features a protrusion pointing toward the tip. At the terminal end of the final antennal segment, there are seven sensilla basiconica and one palmiform sensillum. These sensilla are closely arranged, with the palmiform sensillum positioned laterally to the group of basiconic sensilla.

The antenna of *P. brevitarsis* larvae is relatively robust and consists of four segments. Its surface is smooth and devoid of setae. The segment lengths, from the base to the tip, are 0.73 ± 0.09 mm (*N* = 10), 0.35 ± 0.05 mm (*N* = 10), 0.31 ± 0.03 mm (*N* = 10), and 0.38 ± 0.04 mm (*N* = 10), accounting for approximately 43%, 19%, 19%, and 19% of the total antennal length, respectively (Figure 4B). The first segment is relatively long, while the second and fourth segments are of similar length, and the third segment is shorter with a ventral protrusion near its tip. The front portions of the second and third segments are enlarged, forming an inverted trapezoidal shape. At the tip of the final segment, there are seven short basiconic sensilla.

The maxillary palps of both species are four‐segmented, appearing slender in *A. mongolica* and comparatively thicker in *P. brevitarsis* . The maxillary palps of each species bear a digitiform sensillum on the lateral surface of the distal segment, but their morphologies differ markedly. Cross‐sections of homologous positions reveal that the digitiform sensilla protrude beyond the epidermis in *A. mongolica* (Figure 4C) but are embedded beneath the epidermis in *P. brevitarsis* (Figure 4D).

### 3.4 Morphology Differences of Feeding Apparatus

**FIGURE 5:**
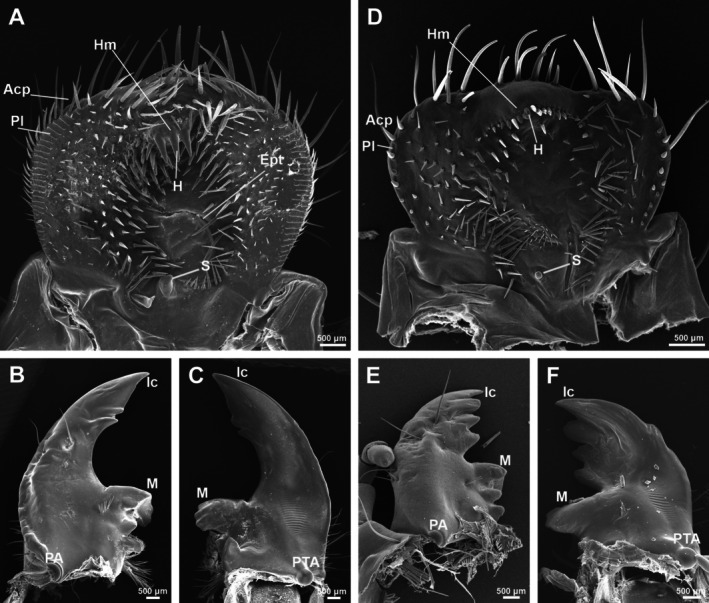
The feeding apparatus of *Anomala mongolica* and *Protaetia brevitarsis*. (A) Epipharynx of *A. maragdina*; (B) Left mandible of *A. mongolica*, dorsal surface; (C) Left mandible of *A. mongolica*, ventral surface; (D) Epipharynx of *P. brevitarsis*; (E) Left mandible of *P. brevitarsis*, dorsal surface; (F) Left mandible of *P. brevitarsis*, ventral surface. Acp, acanthoparia; Ept, epitorma; H, helus; Hm, haptomerum; Ic, incisor; M, molar; PA, preartis; Pl, plegmatium; PTA, postartis; S, sensillum.

The feeding apparatus of white grubs primarily consists of the labrum, epipharynx, a pair of mandibles, and the maxilla‐labial complex. The morphological differences in the feeding organs between the two species are mainly observed in the epipharynx and mandibles (Figure 5).

The larval epipharynx of the two species exhibits significant differences in shape, the apical haptomerum, and the lateral plegmatia (Figure 5A,D). In *A. mongolica* , the epipharynx is nearly fan‐shaped, while in *P. brevitarsis* , it appears generally tri‐lobed (Figure 5D). The haptomerum of *A. mongolica* is equipped with three heli (Figure 5A), whereas that of *P. brevitarsis* features only a row of sensilla basiconica (Figure 5D). The lateral edge of the epipharynx in *A. mongolica* is adorned with plegmatia, comprising 26–28 plegmata, which are interwoven with an equal number of acanthoparia (Figure 5A). In contrast, the lateral edge of the epipharynx in *P. brevitarsis* bears only eight to 10 acanthoparia (Figure 5D).

The mandibles of both species of larvae are highly sclerotized, gradually curving inward at the tip to form an overall triangular shape. The primary difference between the mandibles of the two larvae lies in the morphology of the incisors. In *A. mongolica* , the mandibles feature a blade‐like apical cusp, followed by a smaller cusp posterior to the scissorial notch (Figure 5B,C). In contrast, the incisor regions of *P. brevitarsis* are asymmetrical, with four apical scissorial teeth on the left mandible (Figure 5E,F) and only three teeth on the right mandible.

### 3.5 Morphology Differences of Locomoting Organs

**FIGURE 6:**
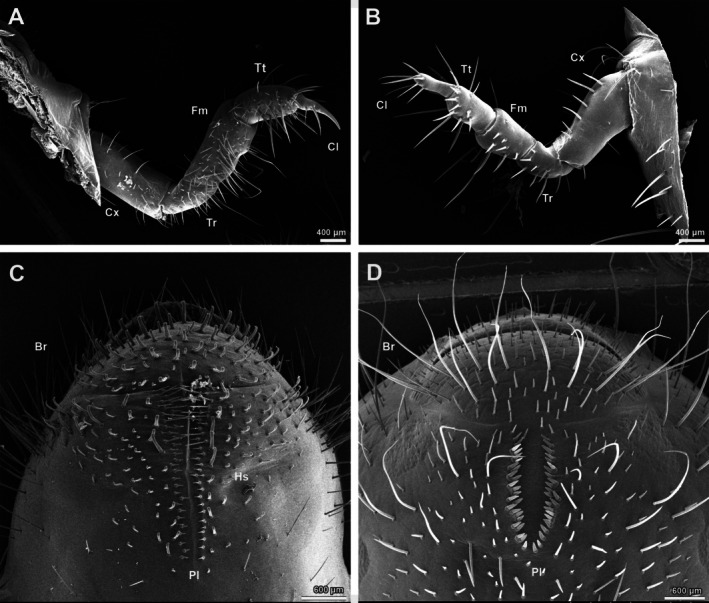
The locomoting organs of *Anomala mongolica* and *Protaetia brevitarsis*. (A) Thoraic legs of *A. mongolica*; (B) Thoraic legs of *P. brevitarsis*; (C) Spiracles of *A. mongolica*; (D) Spiracles of *P. brevitarsis*. Br, barbula; Cl, claw; Cx, coxa; Fm, femur; HS, hamate seta; Pl, palidia; Tt, tibiotarsus; Tr, trochanter.

The thoracic legs of both larvae consist of the coxa, trochanter, femur, tibiotarsus, and claws. The main differences are in the relative lengths of the femur, setal arrangement, and the shape of the claws (Figure 6A,B). The femur and coxa are nearly equal in length in *A. mongolica* (Figure 6A), but the femur is less than half the length of the coxa in *P. brevitarsis* (Figure 6B). The setae on the thoracic legs in *A. mongolica* , especially on the femur and tibiotarsus, are dense, elongated, and extend ventrally (Figure 6A), while in *P. brevitarsis* , they are sparse, short, and point in various directions (Figure 6B). The claws in *A. mongolica* are slender with sharply pointed tips, whereas those of *P. brevitarsis* are thicker with rounded tips.

Both species possess parallel‐arranged palidia at the terminal abdominal segment, but with a slightly different count. A notable distinction is the presence of dense hamate setae in the raster of *A. mongolica* , which are entirely absent in *P. brevitarsis* .

## 4 Discussion

This study is the first attempt to use an improved six‐armed olfactometer to identify the feeding preference differences between two co‐distributed white grubs. Simultaneously, based on scanning electron microscopy, it reveals for the first time the morphological differences between the larvae with different ecological roles, mainly in terms of sensory organs, feeding apparatus, and locomotor organs. These findings provide valuable insights for developing targeted pest management strategies based on species‐specific biological characteristics.

For soil‐dwelling animals that live in dark environments, olfaction serves as the primary sensory mechanism (Matthews and Matthews 2009; Reeves 2011; Eilers et al. 2012). For grubs, the primary olfactory organs are the antennae and labial palps located on the head (Zacharuk and Shields 1991; Scholtz et al. 2004; Weissteiner et al. 2012). Our comparison of the sensory organs between the two species revealed three key differences. First, the terminal three segments of the antennae in *A. mongolica* are longer than in *P. brevitarsis* , indicating enhanced olfactory flexibility for detecting host plant volatiles, as reported in other phytophagous species (Jia et al. 2023; Zhang et al. 2024; Cao et al. 2024). Second, *A. mongolica* possesses more developed sensilla at the antennal tips, which likely enhance chemical detection abilities (Merivee et al. 1999). Third, prominent digitiform sensilla located laterally on the labial palps of *A. mongolica* may function as CO_2_; receptors—a hypothesis supported in other insects, though direct evidence remains limited (Keil 1996; Eilers et al. 2012). The more prominent digitiform sensilla in *A. mongolica* support the hypothesis that these larvae use CO_2_ produced by root respiration to locate host plants during foraging.

The epipharynx and mandibles represent the feeding apparatus components most directly linked to dietary preferences (Zhang 1984; Fang et al. 2018; Li, Fu, et al. 2019; Li, Jiang, and Fang 2019; Qu et al. 2019; Jia et al. 2020, 2021, 2023; Dong et al. 2021; Sun et al. 2024). In this study, the main morphological differences in mouthparts of *A. mongolica* and *P. brevitarsis* are concentrated in the epipharynx and mandibles, specifically in the haptomerum of the epipharynx and the incisor region of the mandibles. The mandibles of *A. mongolica* have blade‐shaped incisors, which help form a cutting edge for severing plant roots, similar to other herbivorous grubs (Gomes et al. 2014; Jia et al. 2023; Zhang et al. 2024; Cao et al. 2024). In contrast, *P. brevitarsis* mandibles lack blade‐shaped incisors and have sharp cusps, similar to other saprophagous, saproxylic, or coprophagous grubs (Micó and Galante 2003; Pereira et al. 2013; Qu et al. 2019; Li, Fu, et al. 2019; Li, Jiang, and Fang 2019; Dong et al. 2021; Sun et al. 2024). These mouthpart characteristics appear consistent across broader taxonomic groups with similar feeding habits. Within Rutelinae, several saprophagous larvae such as *Parastasia brevipes* and *Pelidnota punctata* demonstrate similar mandibular morphology to *P. brevitarsis* , with reduced or absent blade‐shaped incisors and prominent sharp cusps (Ritcher 1966; Neita‐Moreno and Morón 2017; Li, Fu, et al. 2019; Li, Jiang, and Fang 2019; Sun et al. 2024). Similarly, phytophagous larvae of Rutelinae consistently show well‐developed blade‐shaped incisors adapted for cutting plant tissues (Fang et al. 2018; Jia et al. 2023). This morphological pattern extends beyond Rutelinae to other scarab groups, where saprophagous species and phytophagous species show parallel adaptations in mandibular structures (Cao et al. 2024; Jia et al. 2020), suggesting these morphological differences represent convergent adaptations to specific feeding strategies rather than mere taxonomic differences. While the plegmatium also differs between the two larvae, it appears in Rutelinae larvae with different diets (Fang et al. 2018; Sun et al. 2024) and seems less correlated with feeding habits.

The thoracic legs, as the main locomotor organs, exhibit clear morphological differences between the two species with different feeding habits. As observed in our study, *A. mongolica* requires enhanced locomotion to locate host plants in the soil. In contrast, *P. brevitarsis* larvae often emerge from the soil surface and move with their ventral side facing upward, during which the thoracic legs are not involved. The thoracic legs of *A. mongolica* have longer distal segments, well‐developed fossorial setae on the femur, and sharp distal claws, similar to other phytophagous grubs in different groups (Jiang et al. 2019; Jia et al. 2023; Zhang et al. 2024; Cao et al. 2024). In contrast, the thoracic legs of *P. brevitarsis* are shorter, with no fossorial setae on the femur, and its claws are blunt, similar to those of sedentary detritivorous species (Jessop 1986; Li, Fu, et al. 2019; Li, Jiang, and Fang 2019; Sun et al. 2024). Notably, hamate setae, which contribute to grub movement (Jessop 1986; Li, Fu, et al. 2019; Li, Jiang, and Fang 2019; Sun et al. 2024), were present in *A. mongolica* but absent in *P. brevitarsis* .

The six‐armed olfactometer is a classic tool for studying animal behavior in soil (Rasmann et al. 2005), but its original design was too small and transparent for behavior research on white grubs. In this study, we created a larger version using PVC material to better suit larval passage and avoid potential negative phototaxis. This new tool helps us better explore the feeding preferences of grubs. In our experiment, *A. mongolica* preferred containers with plants, while *P. brevitarsis* showed no preference, proving that the six‐armed olfactometer can distinguish larvae feeding preferences. It is worth noting that most (but not all) herbivorous *A. mongolica* larvae prefer containers with plants, likely because odor molecules, including CO_2_, diffuse more easily upward than horizontally. In the future, we plan to seal the container tops with plastic film to allow horizontal gas diffusion, which may highlight more noticeable differences.

White grubs exhibit remarkable diversity in species and habits, making their identification a significant challenge (Ritcher 1966; Zhang 1984; Sawada 1991), particularly when attempting to understand their ecological roles (King 1984; Morón et al. 2018; Ritcher 1966; Zhang 1984). Within the Scarabaeoidea, many closely related species have distinct larval morphologies (Fang et al. 2018; Sun et al. 2024), while larvae from different groups show structural homoplasy due to similar selective pressures (Dong et al. 2021; Sun et al. 2024; Cao et al. 2024). Indeed, homoplasy arising from convergent evolution enables researchers to bypass the need for species identification one by one, providing clues for more efficient inference of the ecological roles of grubs, which has been confirmed in studies of many soil‐dwelling insects (Villani et al. 1999; Jiang et al. 2019). Using an improved soil animal foraging model, our study successfully identified significant morphological differences between two sympatric larval species with distinct feeding habits. These differences in sensory, feeding, and locomotor organs may provide valuable insights for future assessments of the ecological roles of larvae, including grub species.

## Author Contributions

**Yi‐Nuo Chen:** data curation (equal), writing – original draft (equal). **Mei Ying:** data curation (equal), writing – original draft (equal). **Chang Lu:** data curation (equal), investigation (equal). **Lu Jiang:** conceptualization (lead), writing – review and editing (lead).

## Conflicts of Interest

The authors declare no conflicts of interest.

## Supporting information


**Data S1.** ece371925‐sup‐0001‐DataS1.xls.

**Data S2.** ece371925‐sup‐0002‐FigureS1.jpg.

**Data S3.** ece371925‐sup‐0003‐FigureS2.jpg.

**Data S4.** ece371925‐sup‐0004‐FigureS3.gif.

**Data S5.** ece371925‐sup‐0005‐FigureS4.jpg.

**Data S6.** ece371925‐sup‐0006‐FigureS5.png.

**Data S7.** ece371925‐sup‐0007‐FigureS6.jpg.

## Data Availability

Original data were uploaded on the supporting information.
