# Supplementary figures and images for: Consumer or Decomposer? Behavioral and Morphological Diagnosis of White Grubs
Source: Ecol Evol. 2025 Aug 11;15(8):e71925. doi: 10.1002/ece3.71925 (PMC12336419; doi:10.1002/ece3.71925)

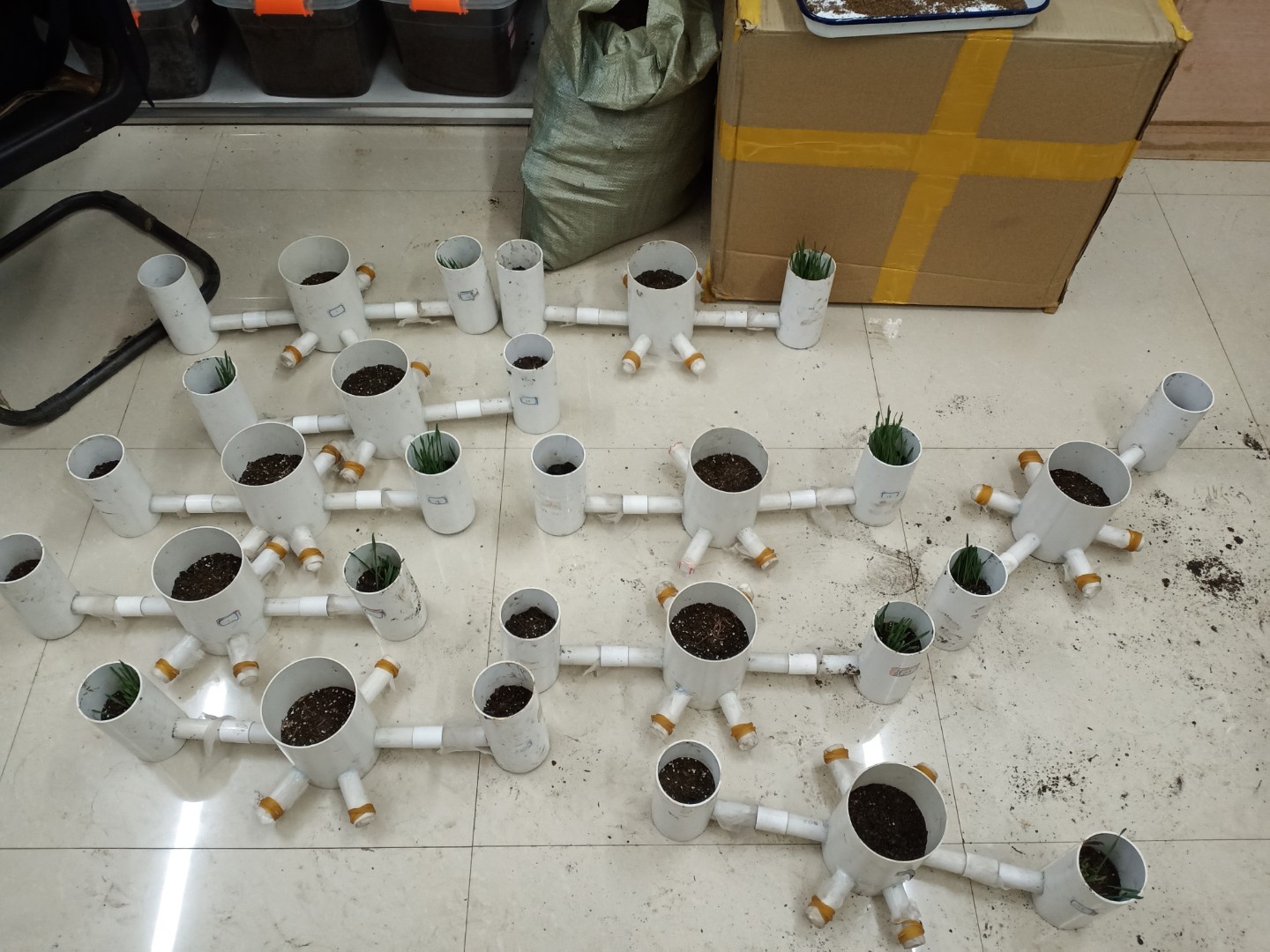

Supplement: Supplementary file 2 — Data S2. ece371925‐sup‐0002‐FigureS1.jpg. [file ECE3-15-e71925-s005.jpg]

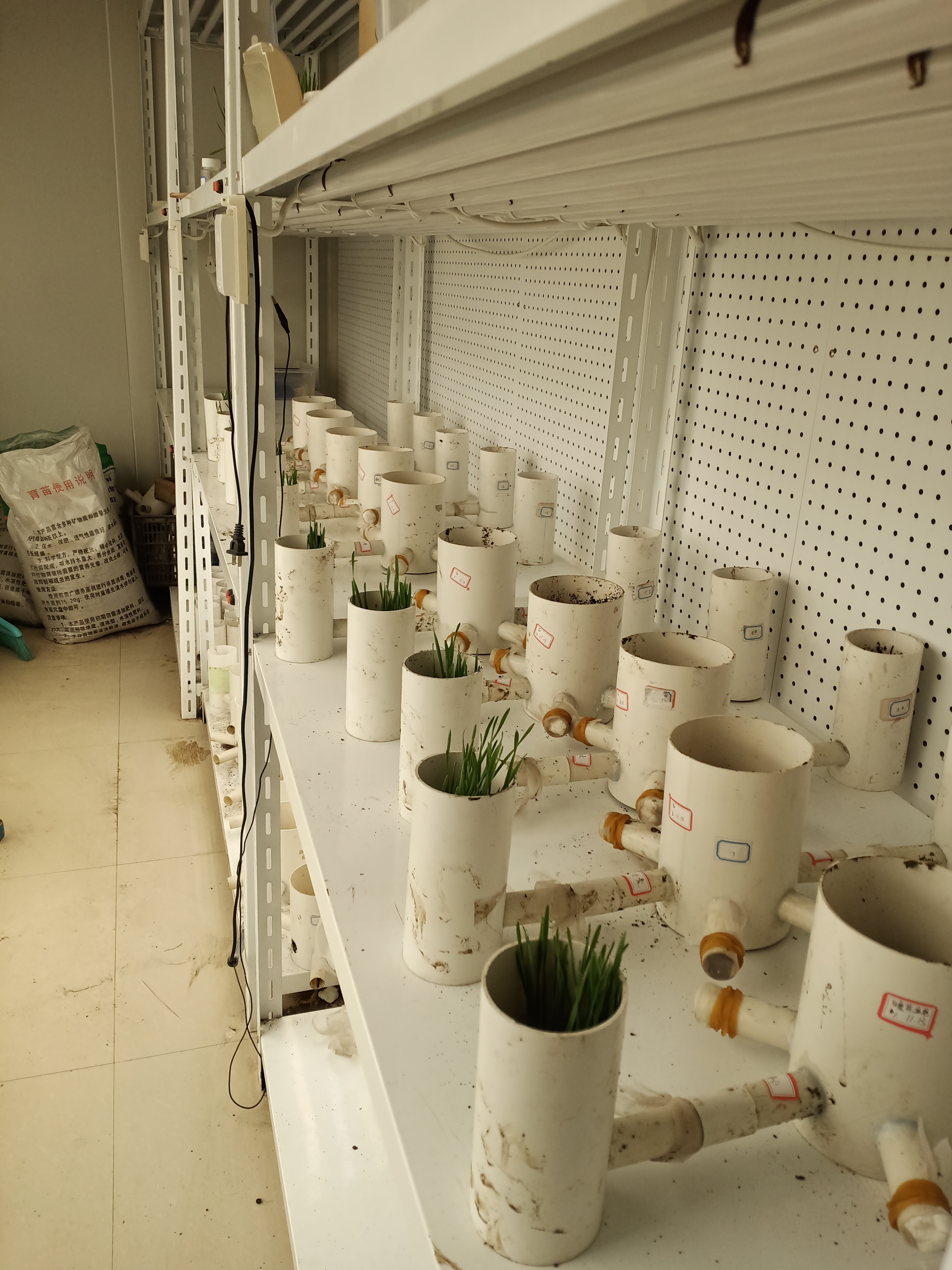

Supplement: Supplementary file 3 — Data S3. ece371925‐sup‐0003‐FigureS2.jpg. [file ECE3-15-e71925-s004.jpg]

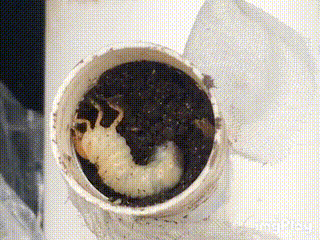

Supplement: Supplementary file 4 — Data S4. ece371925‐sup‐0004‐FigureS3.gif. [file ECE3-15-e71925-s001.gif]

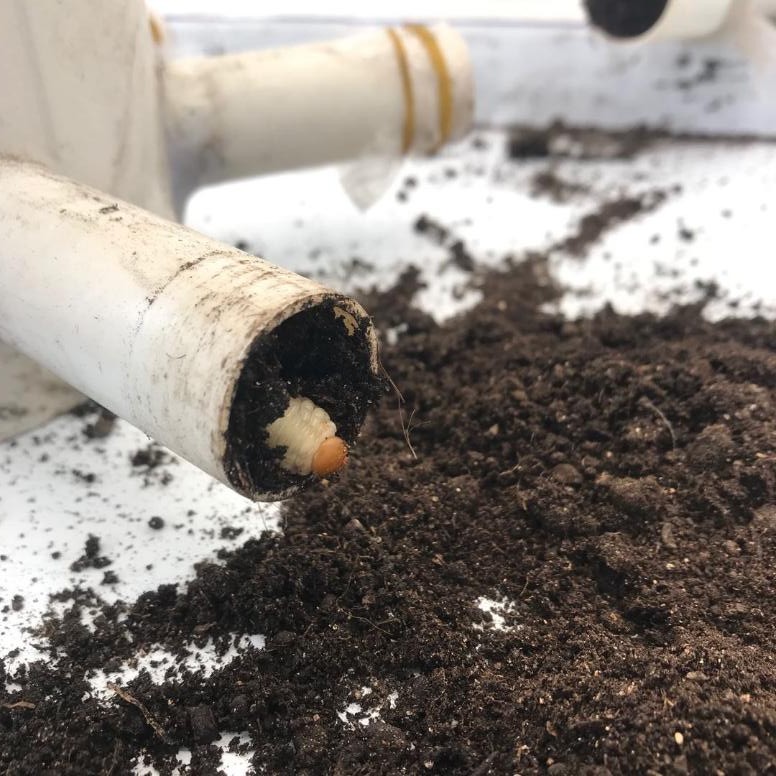

Supplement: Supplementary file 5 — Data S5. ece371925‐sup‐0005‐FigureS4.jpg. [file ECE3-15-e71925-s002.jpg]

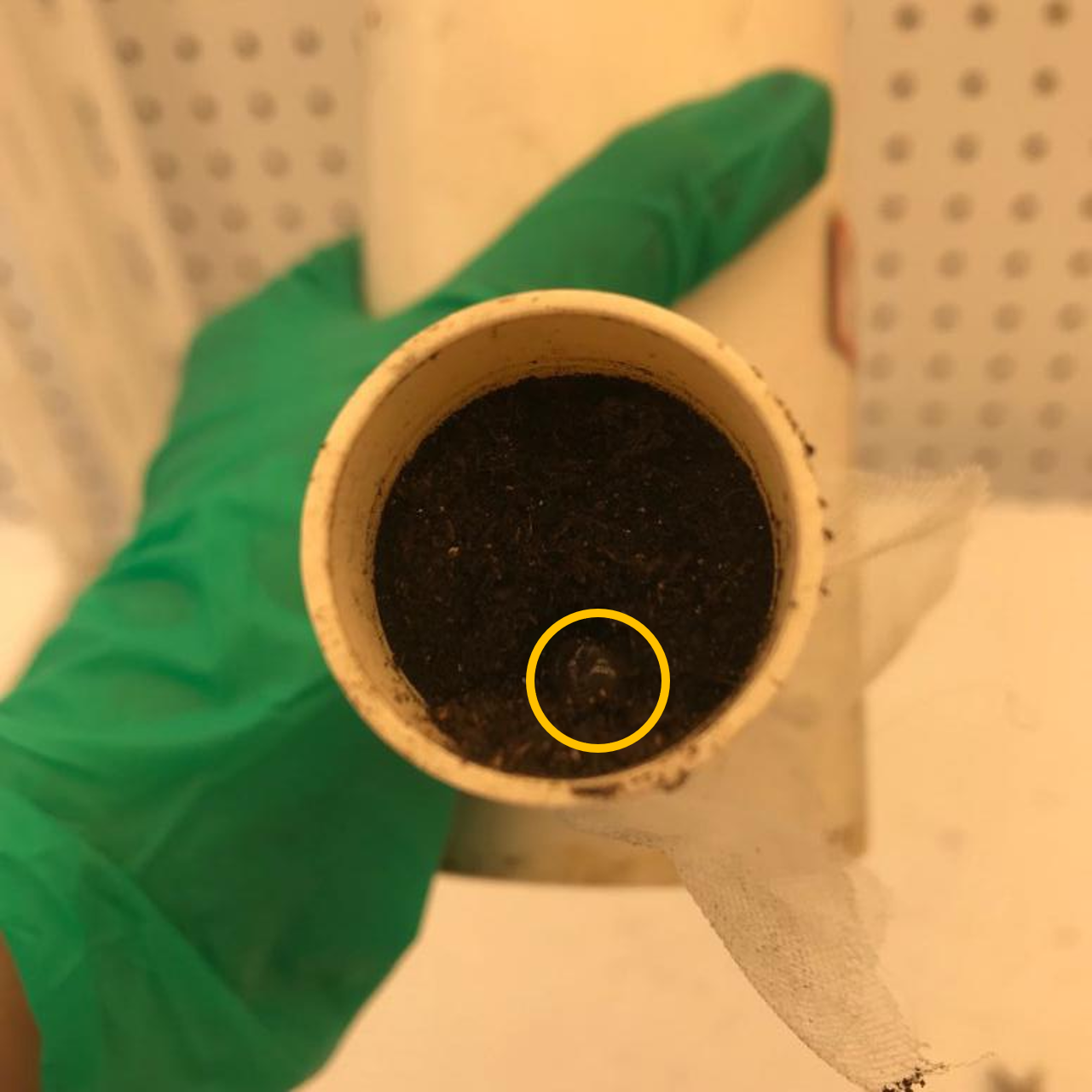

Supplement: Supplementary file 6 — Data S6. ece371925‐sup‐0006‐FigureS5.png. [file ECE3-15-e71925-s006.png]

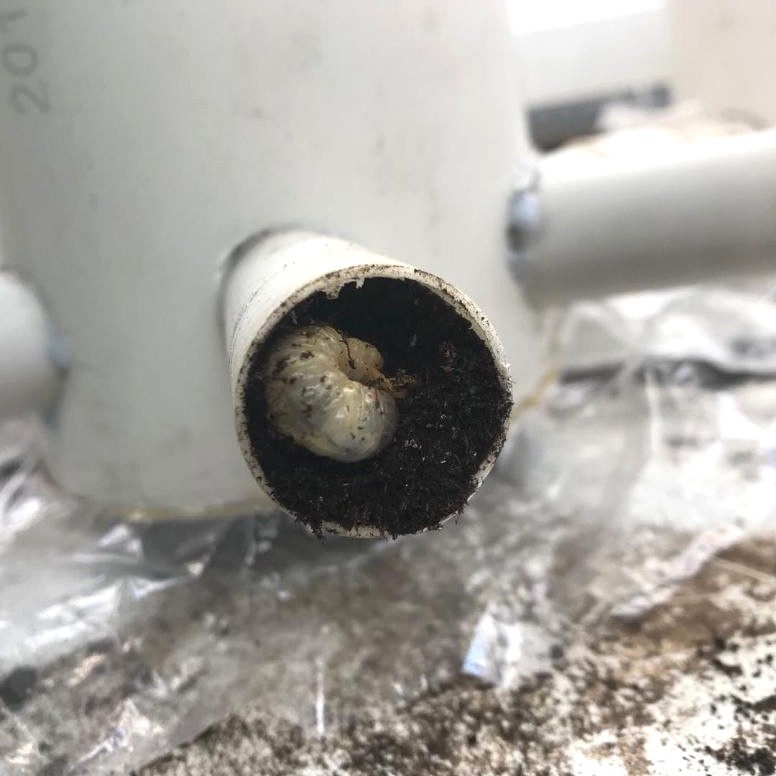

Supplement: Supplementary file 7 — Data S7. ece371925‐sup‐0007‐FigureS6.jpg. [file ECE3-15-e71925-s007.jpg]
